# Microsatellite and mitochondrial DNA analyses unveil the genetic structure of native sheep breeds from three major agro-ecological regions of India

**DOI:** 10.1038/s41598-020-77480-6

**Published:** 2020-11-24

**Authors:** Rekha Sharma, Sonika Ahlawat, Himani Sharma, Priyanka Sharma, Poonam Panchal, Reena Arora, M S Tantia

**Affiliations:** ICAR-National Bureau of Animal Genetic Resources, Karnal, India

**Keywords:** Genetics, Molecular biology

## Abstract

Sheep farming has been fundamental to many civilizations in the world and is practiced in India since antiquity. Several thousand years of adaptation to local environmental conditions and selective breeding have evolved 44 sheep breeds in India. They are paramount in terms of economic, scientific, and cultural heritage. Genetic characterization information is imperative for sustainable utilization and conservation of ovine heritage. In this study, the genetic diversity, differentiation, and structure of 11 indigenous sheep breeds from three different agro-ecological zones of India were explored with genomic microsatellite loci and mitochondrial DNA (D loop). The estimated diversity parameters indicated that populations retained high levels of genetic diversity (Na = 8.27 ± 0.17; Ho = 0.65 ± 0.01), which provides an optimistic viewpoint for their survival. However, significant inbreeding was also observed in the nine populations. Moderate genetic differentiation existed among the groups (F_ST_ = 0.129 ± 0.012), and most likely clusters existing in the dataset are seven. Phylogenetic clustering was in line with the geographical locations of sheep populations. Mitochondrial sequences revealed high haplotype diversity with the existence of maternal haplogroups A, B, and C, and signals of population expansion. Decreased genetic diversity and unique maternal lineage (C) in endangered Tibetan and Bonpala sheep breed, warrant their immediate scientific management. Overall, the quantitative data reported here on the extant variability, and genetic relationships among the Indian sheep breeds, provide critically important inputs that will be valuable for the decision-making process on their management, both for the conservation of endangered breeds, and formulation of breeding programs to check genetic erosion.

## Introduction

Sheep, *Ovis aries*, are a highly versatile and adaptable species. From their domestication in the Fertile Crescent, approximately 11,000 years ago, sheep now span the sundry terrains of all inhabited continents and are utilized as a source of food (meat, milk, fat) and clothing (skin, wool)^1^. India has the second-largest sheep inventory in the world (6.4%) after China, with 74.26 million heads, and sheep number has increased (14.13%) in the recent past (2012 to 2019)^2^. Sheep are an important species of livestock for India. Sheep contribute immensely to the agrarian economy and to the livelihoods of small and marginal farmers in the harshest areas, where agriculture and dairying are not cost-effective. Mountains from Afganistan to Armenia have various wild varieties of sheep (*Ovis orientalis vignai*), who were possibly the ancestors of Indian sheep^3^. Urial and Argali stock are considered to be the progenitor of Indian sheep^3^. Thousands of years of natural selection for adaptation to varied agro-ecological conditions^4^, and planned field level breeding for environmental tolerance, behavioral, and commercial traits are reflected in the form of 44 sheep breeds in India (www.nbagr.res.in).

Genetic diversity (the variation of alleles and genotypes present in a population) reflects population size, history, ecology, and adaptability^5^. It plays a vital role in providing traits responsible for improvement, survival, and adaptation of a species^6^. Future challenges, such as climate change, emerging diseases, pressure on land and water, and shifting market demands, make it even more important to ensure that livestock genetic resources are conserved and used sustainably^7^. The vast Indian sheep biodiversity is being eroded rapidly, with more than 50% of sheep breeds currently under threat^8^. Thus, a better understanding of the indigenous sheep genetic diversity can pave way for devising conservation strategies; facilitate their effective use for future breeding programs and, formulating management policies. Genetic diversity is often assessed by molecular markers, including the nuclear microsatellites, also referred to as the simple sequence repeat markers (nSSR) and mitochondrial DNA (mtDNA)^9^. Nuclear SSR has been widely accepted as a useful tool for measuring genetic diversity and divergence within and among livestock populations^10^. Microsatellite markers are consistent with Mendel’s laws of inheritance, are highly polymorphic, and generally have the co-dominant inheritance. Moreover, these are highly reproducible and easy to perform^11^. Therefore, genetic diversity data obtained by nSSR is used for surveying the genetic variability of livestock species that provides theory references to prevent the loss of genetic diversity, over the time^12^. These are being used to estimate the diversity of autochthonous sheep breeds all over the world^13,14^, including India^8,15^.

Mitochondrial DNA has contributed immensely to elucidating the molecular phylogeny of many livestock species due to a higher mutation rate than the nuclear DNA, maternal inheritance, lack of recombination, and existence as multiple copies. The control region or the displacement region (D-loop) has particularly attracted the attention of researchers, to analyze phylogenetic relationships, and explore domestication events across species^16,17^. Multiphyletic origins involving 5 phylogenetically divergent mtDNA haplogroups (A–E) have been delineated in sheep, with haplogroups A and B being recorded in many countries and continents across the globe, while for haplogroups C, D, and E, the geographic distribution is relatively less widespread^18^. Studies on Indian domestic sheep have unveiled that haplogroup A is mainly represented in indigenous breeds, followed by haplogroup B^19^. Haplogroup C, which is considered a less frequent haplogroup worldwide, has also been seen in < 1% of the sampled animals, in one study on Indian sheep breeds^20^.

The sheep breeds in India have been classified into four groups, based on agro-ecological regions^3^, that is (1) North temperate region (NT), (2) North-Western arid and semi-arid region (NWASA), (3) Southern peninsular region (SP), and (4) Eastern region (ET)^4^. Himalaya mountains constitute the NT region. The NWASA consists of scattered hills in vast alluvial plains and an undulating landscape of the sandy desert. This is the primary region in the country for carpet-wool production and has the second largest population of sheep among the four regions. The SP region has the largest sheep population in the country, and these are maintained primarily for meat. The zone has a hot and humid coastline and semi-arid central peninsular region. Its topography includes sandy tracts, mountain ranges, plateaus, deltas, planes and, even highlands. The ET region is primarily hot and humid, apart from a few sub-temperate and humid areas of eastern states. The landscape is highly variable with alluvial plains along with undulations, plateaus and table-land, and Himalaya hill ranges as well as valleys in the north-eastern area. Here, sheep are primarily maintained for meat and produce extremely coarse and hairy fleece (https://www.dahd.nic.in).

The adaptation of different Indian breeds to a wide range of agroecology offers the necessary variability, that provides the opportunity to meet the future demands for food, and endows with the flexibility to respond to altered markets and needs^5^. So far, the characterization of sheep diversity in India has largely been explored using microsatellites, with limited studies utilizing mitochondrial DNA. These are mainly restricted to a single population^21^, with very few studies targeting more number of breeds^22,23^. Genetic variation and relationships among the majority of recognized sheep breeds of NWASA region have been established using microsatellite markers^24^. Comprehensive knowledge about the sheep breeds of the remaining three agro-ecological regions of India is still sought after, for designing suitable and sustainable sheep breeding programs and conservation strategies.

**Figure 1:**
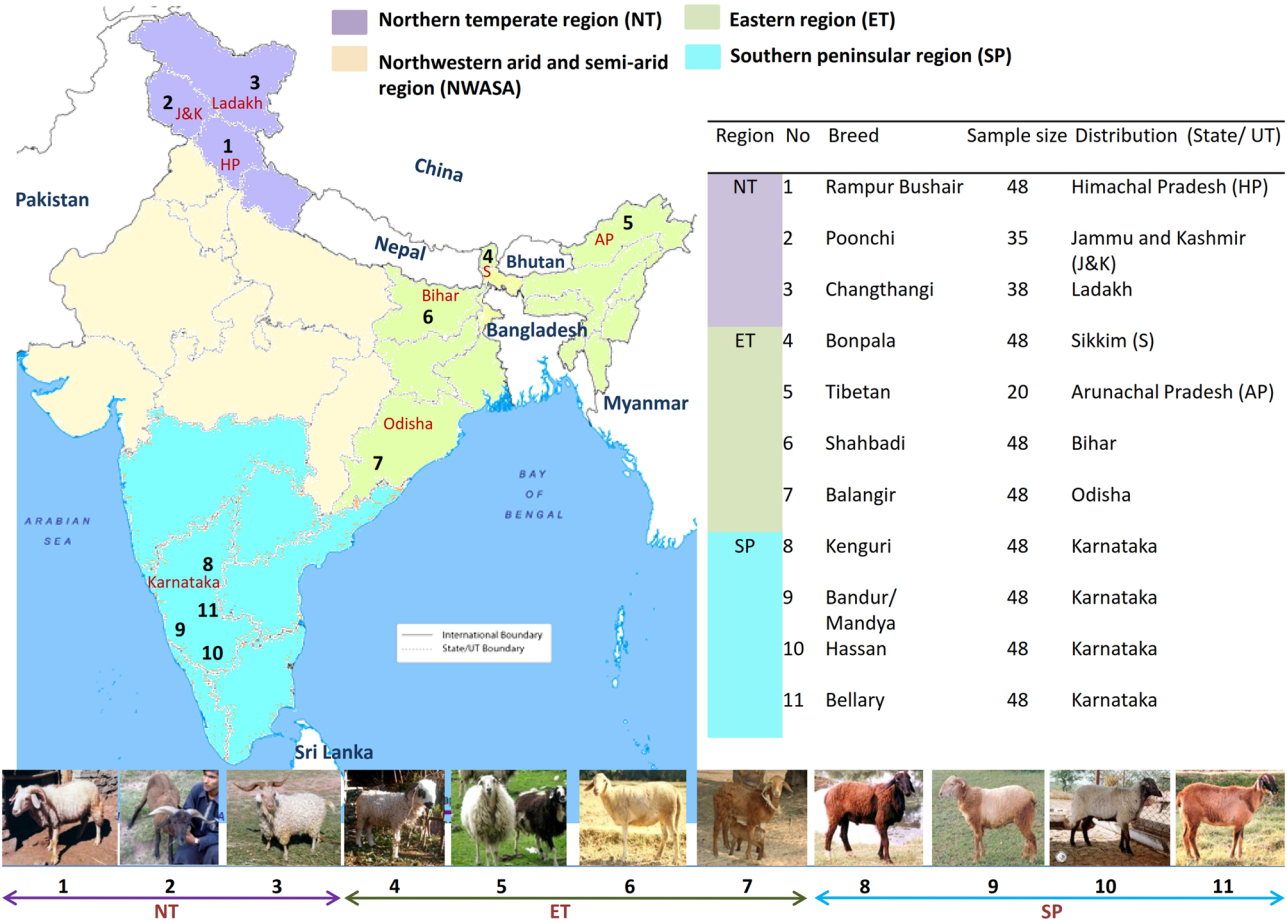
Distribution of Indian sheep breeds included in the study.

The present study was undertaken to assess the genetic diversity, population structure, and phylogeography of 11 unexplored indigenous sheep breeds from three agro-ecological regions (NT, SP, ET) of the country (Fig. 1), using both nSSR and mtDNA markers. Results obtained using two types of markers that have different modes of inheritance, and degrees of polymorphism will provide inclusive insight into the lineage relationship of various Indian sheep breeds, and molecular information on the genetic variability existing within and among them. It will act as a baseline data to devise strategies for sustainable development of precious Indian sheep resources.

## Results

### Mitochondrial DNA sequence variation and genetic diversity in Indian sheep breeds

In this study, we amplified a 1246 bp fragment of ovine mitochondrial control region encompassing part of 12S rRNA coding region and tRNA^Phe^ gene. Analysis of the amplified sequences was restricted to a 1,059 bp fragment, that corresponds to a consensus region defined by our sequences and those retrieved from the GenBank (10 reference sequences), representing 5 well-defined sheep haplogroups. Sequence analysis yielded a total of 83 novel haplotypes in Indian sheep, with overall haplotype diversity (Hd) value of 0.8502 ± 0.024, and nucleotide diversity (π) equal to 0.00373 ± 0.00029. The sequences were defined by 26 singleton variable sites and 44 parsimony informative sites. The maximum number of haplotypes was observed in Bonpala and Poonchi sheep, and minimum in Tibetan sheep. The Hd values ranged from 0.7526 ± 0.099 in Changthangi to 0.9004 ± 0.056 in Poonchi. The estimates of nucleotide diversity (π) were maximum for Tibetan sheep and minimum for Bandur sheep. Detailed statistics summarizing various genetic diversity parameters are given in Table 1. Out of 83 haplotypes, 16 were shared within breeds, and 10 were common among breeds. The majority of haplotypes (57) were private to individual samples, thus suggesting high variation in the analyzed region of the mtDNA. The most frequent haplotype was present in 84 animals, from all the breeds studied, and the second most frequent haplotype was shared by 14 samples from 8 breeds.

**Table 1:** Genetic diversity indices of 11 Indian sheep breeds based on mitochondrial D loop sequence.

Region	Breed/Population	N	H	Hd	π	Number of haplotypes in haplogroups		
						HA	HB	HC
NT	Rampur Bushair	20	8	0.8053 ± 0.070	0.00408 ± 0.00076	5	3	–
	Poonchi	22	14	0.9004 ± 0.056	0.00423 ± 0.00073	9	5	–
	Changthangi	20	9	0.7526 ± 0.099	0.00355 ± 0.00079	6	3	–
ET	Bonpala	20	14	0.8895 ± 0.068	0.00392 ± 0.00093	13	–	1
	Tibetan	20	6	0.7632 ± 0.066	0.00607 ± 0.00081	3	2	1
	Shahbadi	20	11	0.8421 ± 0.077	0.00329 ± 0.00082	8	3	–
	Balangir	20	9	0.8158 ± 0.073	0.00211 ± 0.00039	9	–	–
SP	Kenguri	20	10	0.8684 ± 0.063	0.00301 ± 0.00077	9	1	–
	Bandur	20	8	0.8263 ± 0.061	0.00153 ± 0.00028	8	–	
	Hassan	20	12	0.8474 ± 0.079	0.00285 ± 0.00069	11	1	
	Bellary	20	10	0.8316 ± 0.075	0.00170 ± 0.00030	10	–	
	Overall values	222	83	0.8502 ± 0.024	0.00373 ± 0.00029	66	15	2

N = Sample size; H = Number of haplotypes; Hd = Haplotype diversity; π = Nucleotide diversity; HA = Haplogroup A; HB = Haplogroup B; HC = Haplogroup C.

### mtDNA based phylogenetic analysis and genetic structure of Indian sheep

**Figure 2:**
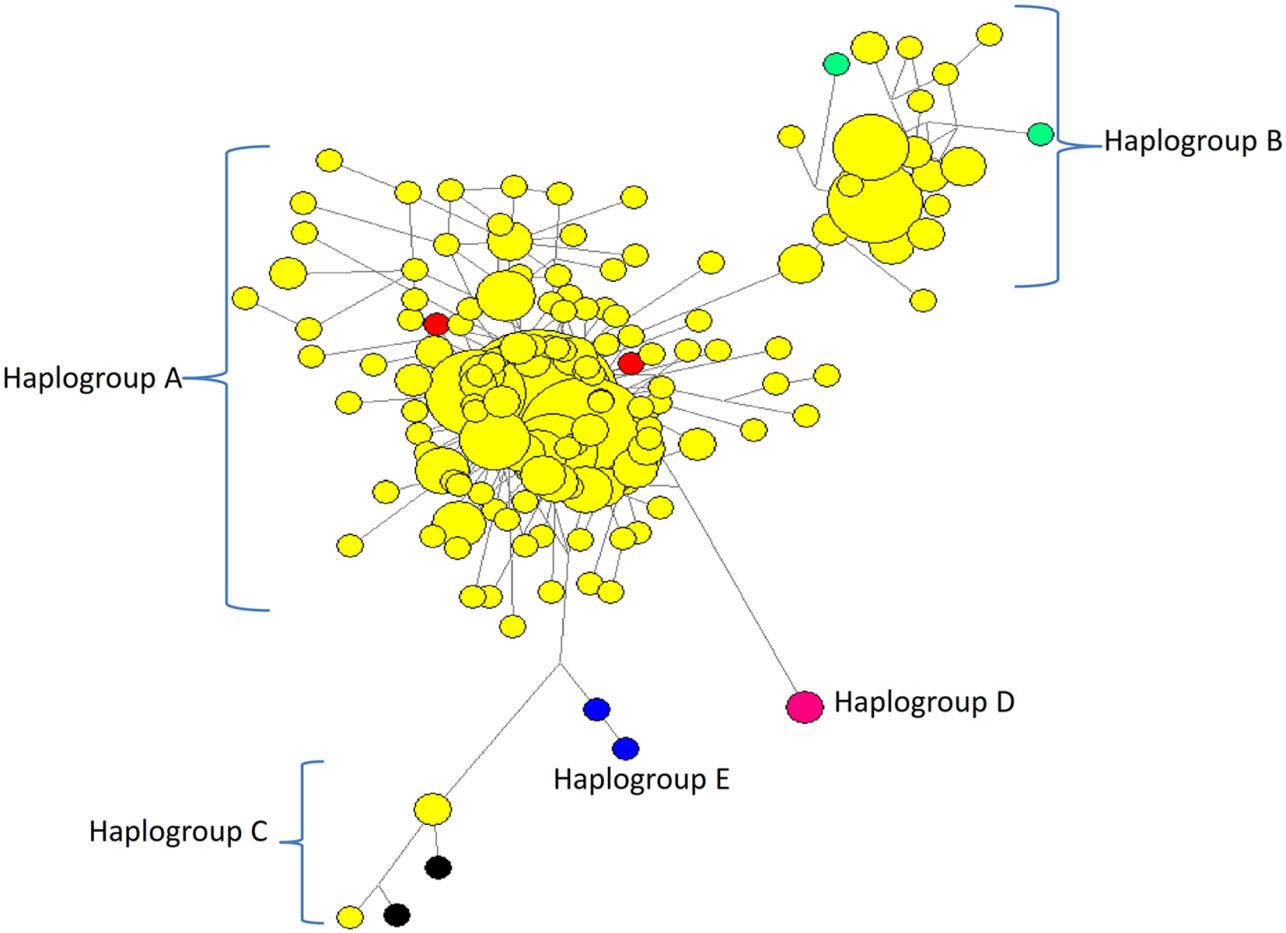
Median joining network constructed using NETWORK 10.0.0^47^ (https://www.fluxus-engineering.com/) showing the phylogenetic relationship of Indian sheep with 5 established haplogroups reported worldwide (Haplogroups A-E are highlighted with different colors). Indian sheep haplotypes are depicted in yellow color. The size of the circle is proportional to haplotype frequency.

A median joining (MJ) network constructed using the haplotypes from Indian sheep breeds along with 10 reference sequences for the 5 known sheep haplogroups, revealed that Indian sheep were undoubtedly separated into three distinct clusters that corresponded to haplogroups A, B, and C. Out of a total of 154 Indian sheep specific haplotypes, 134 grouped with haplogroup A, 18 with haplogroup B and 2 with haplogroup C (Fig. 2). The phylogenetic topology obtained by the construction of neighbor joining (NJ) tree was also similar (Supplementary Fig. S1). The proportion of animals in haplogroups A, B, and C was 88.86%, 10.46%, and 0.66%, respectively. In addition to nine breeds that previously showed conformity to haplogroup B (Marwari, Nali, Kheri, Muzaffarnagri, Sonadi, Chokla, Jaisalmeri, Patanwadi and Garole), this study revealed that seven more breeds had their representation in haplogroup B (Table 1). Interestingly, unlike other breeds, haplogroup B was the most frequent haplogroup in Tibetan sheep. Our study, for the first time, reports clustering of some animals of breeds of the Eastern region i.e. Bonpala and Tibetan with haplogroup C. From the MJ network profile, a star-like shape for the haplogroup A could be appreciated, since the most common haplotype comprising of 166 members was present at the center, and was surrounded by haplotypes that were separated by few mutations only. If we specifically discuss the 11 sheep breeds analyzed in this study, out of a total of 16 haplotypes that were shared within breeds, the number of haplotypes that belonged to haplogroups A, B, and C were 10, 5, and 1, respectively. Among the 10 haplotypes that were common among breeds, 8 conformed to haplogroup A, and 2 represented haplogroup B.

Assessment of mismatch distribution in Indian sheep breeds revealed Raggedness index of 0.012 (*P* = 0.97), 0.043 (*P* = 0.85), 0.013 (*P* = 0.76) and 0.010 (*P* = 1.00), respectively, in sheep breeds from NT, NWASA, SP and ET regions, hinting at demographic expansion. Additionally, the sum of squared deviations (SSD) between the observed and expected mismatch values also presented non-significant low values, again suggesting population expansion. This observation was also substantiated by the results of Fu’s Fs and Tajima’s D neutrality tests. Significant negative Fu’s Fs values, which are inferred as signs of either purifying selection or demographic expansion were evident in all groups. Similarly, Tajima’s D value was negative, but significant only in the case of sheep from ET and SP zones of India. Details of population demographic parameters based on analysis of the mtDNA D loop region in Indian sheep are presented in Table 2.

**Table 2:** Population demographic parameters and neutrality tests for Indian sheep populations.

Parameter	Northern temperate (NT)	North-western arid and semi-arid (NWASA)	Southern Peninsular (SP)	Eastern (ET)
Sample size (N)	62	102	175	110
Segregating sites (S)	33	22	60	66
Sum of squared deviations (SSD) (*P* value)	0.0128 (0.78)	0.0218 (0.77)	0.0001 (0.97)	0.0073 (0.70)
Raggedness index (*P* value)	0.1234 (0.97)	0.0432 (0.85)	0.0133 (0.76)	0.0103 (1.00)
Fu’s F_s_ (*P* value)	− 13.67 (0.00)	− 12.28 (0.00)	− 26.12 (0.00)	− 25.66 (0.00)
Tajima’s D (*P* value)	− 1.32 (0.06)	− 0.97 (0.15)	− 2.10 (0.00)	− 2.06 (0.00)

### Genetic variability in Indian sheep breeds based on microsatellite markers

The genotype data generated in the present study showed that a significant amount of genetic variation is maintained in the Indian sheep populations. All the markers were found to be polymorphic in each of the 11 analyzed populations. Genotype inferring errors due to stuttering, large allele dropout, and high frequency of null alleles were not detected. Non-significant linkage disequilibrium was ascertained between these loci thus, all the loci were retained for diversity and differentiation analysis. The level of variation depicted by the number of alleles at each locus serves as a measure of genetic variability. FAO has specified a minimum of four different alleles per locus, for evaluation of genetic differences between the breeds^10^. By this criterion, all the 25 microsatellite loci showed ample polymorphism for evaluating within breed genetic variability, and for exploring the genetic differences (Table 3). A total of 517 alleles, with an overall mean of 8.27 ± 0.19 alleles per locus, were observed in the 11 sheep breeds. The most polymorphic loci were OarCP49 and BM1314, with 13 alleles, while BM6506 was observed to be the least polymorphic with 4 alleles across the 11 sheep breeds.

**Table 3:** Mean diversity indices, F-statistics (F_IS_, F_IT_, F_ST_), and gene flow (Nm) values across 25 microsatellite markers in 11 Indian sheep breeds.

Locus	Na	Ne	I	Ho	He	F	Ar	F_IS_	F_IT_	F_ST_	Nm
BM0757	5	3.55	1.34	0.63	0.69	0.08	6.66	0.09	0.20	0.12	1.81
BM0827	7	3.36	1.39	0.64	0.68	0.06	6.75	0.05	0.15	0.10	2.28
BM1314	13	6.74	2.06	0.67	0.81	0.20	15.17	0.18	0.28	0.13	1.69
BM6506	4	1.95	0.78	0.46	0.48	0.04	4.04	0.04	0.27	0.24	0.78
BM6526	9	4.50	1.66	0.76	0.75	− 0.01	8.79	− 0.01	0.11	0.12	1.82
BM8125	7	2.45	1.13	0.51	0.53	0.07	6.61	0.05	0.13	0.08	2.95
CSRD247	9	4.81	1.71	0.72	0.77	0.06	10.26	0.07	0.19	0.13	1.68
CSSM31	11	6.00	1.97	0.72	0.81	0.09	15.21	0.10	0.22	0.13	1.67
CSSM47	7	2.07	0.91	0.32	0.43	0.28	6.64	0.25	0.51	0.35	0.46
HSC	9	5.35	1.81	0.75	0.80	0.07	9.65	0.07	0.13	0.07	3.36
INRA63	10	5.10	1.85	0.80	0.79	− 0.02	10.40	− 0.01	0.06	0.07	3.41
MAF214	8	4.04	1.56	0.66	0.73	0.09	10.67	0.10	0.22	0.13	1.64
OarAE129	7	3.20	1.34	0.51	0.66	0.23	8.72	0.23	0.37	0.19	1.08
OarCP20	8	2.88	1.34	0.69	0.63	− 0.10	8.82	− 0.10	0.05	0.13	1.65
OarCP34	7	3.81	1.49	0.65	0.72	0.10	7.97	0.11	0.20	0.10	2.26
OarCP49	13	7.16	2.10	0.79	0.83	0.04	14.09	0.04	0.13	0.10	2.27
OarFCB128	7	3.76	1.49	0.72	0.71	− 0.03	8.65	− 0.01	0.13	0.13	1.62
OarFCB48	10	5.17	1.87	0.67	0.79	0.16	10.78	0.16	0.23	0.09	2.49
OarHH35	8	4.39	1.67	0.69	0.75	0.06	10.07	0.07	0.20	0.13	1.67
OarHH41	9	3.88	1.60	0.66	0.73	0.10	8.17	0.10	0.18	0.09	2.39
OarHH47	11	5.63	1.94	0.80	0.81	0.02	10.96	0.02	0.08	0.06	3.73
OarHH64	7	3.24	1.33	0.40	0.64	0.35	8.06	0.37	0.49	0.19	1.10
OarJMP029	9	4.26	1.63	0.75	0.74	− 0.01	9.25	− 0.01	0.09	0.10	2.20
OarJMP08	9	4.69	1.74	0.74	0.78	0.05	9.26	0.05	0.14	0.10	2.25
OarVH72	6	3.27	1.35	0.60	0.68	0.12	7.26	0.11	0.23	0.13	1.61
Mean	8.27	4.21	1.56	0.65	0.71	0.08	9.31	0.08	0.20	0.13	2.00
SE	0.19	0.11	0.03	0.01	0.01	0.01	0.53	0.02	0.02	0.01	0.16

Na = Observed number of alleles; Ne = Effective number of alleles; I = Shannon's information index; Ho = Observed heterozygosity; He = Expected heterozygosity; F = Heterozygore deficiency/ inbreeding coefficient; Ar = Allelic richness; F_IS_, F_IT_, F_ST_ = F-statistics values according to Weir and Cockerham^50^.

The average observed number of alleles per locus ranged from 5.96 in Tibetan and Bonpala to 9.12 in Kenguri and Rampur Bushair sheep (Table 4). The average effective number of alleles in a population varied from 2.99 (Bonpala) to 4.72 (Shahbadi), with a mean across all the loci of 4.21 ± 0.11. Lower values of the expected number of alleles, as compared to the observed number of alleles in all the populations, suggested that there were many low-frequency alleles in the populations. The private alleles, confined to one population only, were detected, but most of them were rare alleles having less than 5% allele frequencies. All the microsatellite markers were highly polymorphic according to the mean value of Shannon’s information index (I). Estimates of observed heterozygosity across all the loci and populations (0.65 ± 0.01), confirmed the remarkable level of diversity existing in the Indian sheep. Observed heterozygosity was lower than the expected heterozygosity in all the populations, except for the Poonchi and Bonpala. Similarly, analysis of F_IS_ evidenced significant (*P* < 0.05) heterozygote deficiency in all the populations, except for the Poonchi and Bonpala, which on the contrary depicted heterozygote excess (Table 4).

**Table 4:** Genetic diversity indices of 25 microsatellite markers in 11 populations of sheep in India (See Fig. 1 for the location of each population).

Region	Sheep breed	Total alleles	Na	Ne	I	Ho	He	uHe	F	Ar
NT	Rampur Bushair	228	9.12	4.49	1.66	0.66	0.74	0.74	0.11*	7.21
	Poonchi	214	8.56	4.54	1.69	0.77	0.76	0.77	− 0.01	7.35
	Changthangi	219	8.76	4.52	1.64	0.68	0.72	0.72	0.06*	7.28
ET	Bonpala	149	5.96	2.99	1.24	0.64	0.63	0.64	− 0.02	4.84
	Tibetan	149	5.96	3.92	1.42	0.52	0.68	0.70	0.23*	5.82
	Shahbadi	223	8.92	4.72	1.68	0.65	0.74	0.75	0.13*	7.23
	Balangir	205	8.2	4.22	1.55	0.65	0.71	0.72	0.09*	6.55
SP	Kenguri	228	9.12	4.46	1.61	0.67	0.72	0.72	0.07*	6.99
	Bandur	218	8.72	3.75	1.49	0.61	0.68	0.69	0.11*	6.58
	Hassan	217	8.68	4.23	1.59	0.68	0.71	0.72	0.04*	6.88
	Bellary	224	8.96	4.49	1.64	0.65	0.73	0.74	0.10*	7.07
	Mean	206.73	8.27	4.21	1.56	0.65	0.71	0.72	0.08*	9.31
	SE	8.42	0.19	0.11	0.03	0.01	0.01	0.01	0.01	0.53

Na = Number of alleles; Ne = Number of effective alleles; I = Shannon’s information index; Ho = Observed heterozygosity; He = Expected heterozygosity; uHe = Unbiased expected heterozygosity; F = Heterozygote deficiency (inbreeding coefficient); Ar = Allelic richness. *P < 0.05.

### nSSR based genetic differentiation and population structure

To describe the level of heterogeneity within and between the Indian sheep populations, F-statistics values were determined. Results for each of the 25 loci, across all the populations, are presented in Table 3. The global deficit of heterozygotes across populations (F_IT_) amounted to 20%. An overall significant (*P* < 0.05) deficit of heterozygotes (F_IS_) of 8.5% occurred in the analyzed loci because of inbreeding within the populations. There was a moderate (0.05 < Fst < 0.15) breed differentiation between the 11 populations. The multi-locus F_ST_ value of breed differentiation indicated that 12.9% of the total genetic variation was due to the unique allelic differences between the breeds. The remaining 87.1% corresponded to the differences among the individuals of a breed, across the 25 markers. Pair-wise F_ST_ coefficients between the two populations are shown in Table 5, above diagonal. All pair-wise F_ST_ values were significant (*P* < 0.05). In summary, it revealed the least differentiation between the Bellary-Hassan populations of the SP region. The highest divergence was observed between Changthangi (NT) and Bandur (SP) and was closely followed by Tibetan (ET) and Bandur (SP). The average estimated effective number of migrants per generation, between pairs of the populations (Supplementary Table S1), was of moderate magnitude (Nm = 2.0 ± 0.16). The highest gene flow was observed among Bellary and Hassan (Nm = 22.55) populations of the SP region. Similarly, genetic variation among the different groups, compared using AMOVA (analysis of molecular variance), revealed that there was an 11.1% variation among the populations (Supplementary Table S2), which is consistent with the F_ST_ results (12.9%). The pair-wise fixation index (F_ST_) value provided by AMOVA through 99 permutations differed significantly from zero at *P* < 0.05. The largest genetic distance (D_A_) was recorded between Changthangi (NT) and Bandur (SP) sheep breeds, while Bellary and Hassan of the SP region were closest.

**Table 5:** Pair-wise population matrix of Nei’s genetic distance (below diagonal) and F_ST_ values (above diagonal) among Indian sheep breeds.

1	2	3	4	5	6	7	8	9	10	11	
–	0.033	0.085	0.086	0.110	0.054	0.038	0.041	0.063	0.052	0.055	1
0.216	–	0.079	0.091	0.095	0.063	0.049	0.046	0.070	0.051	0.051	2
0.618	0.592	–	0.117	0.118	0.085	0.096	0.099	0.127	0.112	0.105	3
0.538	0.598	0.759	–	0.057	0.081	0.095	0.093	0.114	0.092	0.089	4
0.927	0.763	0.942	0.943	–	0.115	0.115	0.110	0.126	0.105	0.099	5
0.389	0.500	0.661	0.508	0.935	–	0.059	0.054	0.065	0.055	0.052	6
0.231	0.316	0.652	0.571	0.899	0.387	–	0.028	0.056	0.038	0.043	7
0.253	0.302	0.688	0.562	0.842	0.352	0.131	–	0.040	0.026	0.028	8
0.393	0.470	0.950	0.613	0.933	0.398	0.332	0.230	–	0.036	0.042	9
0.334	0.344	0.812	0.534	0.772	0.343	0.217	0.102	0.196	–	0.011	10
0.384	0.373	0.819	0.542	0.756	0.355	0.273	0.165	0.241	0.058	–	11

NT region: 1 = Rampur Bushair, 2 = Poonchi, 3 = Changthangi; ET Region: 4 = Bonpala, 5 = Tibetan, 6 = Shahbadi, 7 = Balangir; SP region: 8 = Kenguri, 9 = Bandur, 10 = Hassan, 11 = Bellary.

**Figure 3:**
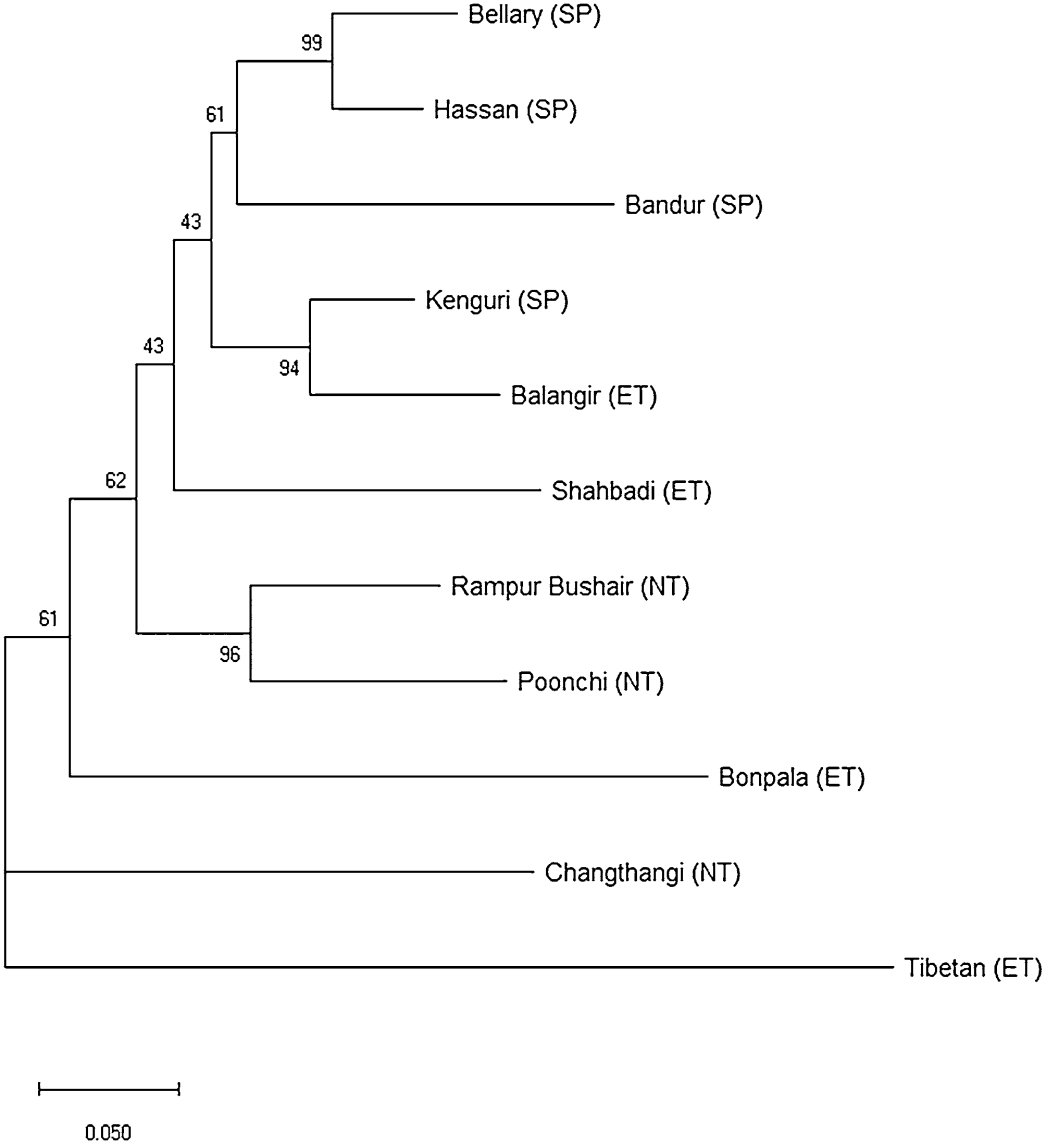
Neighbour Joining (NJ) dendrogram based on pair-wise Da distance using MEGA 6^42^ (https://www.megasoftware.net/). Values at nodes indicate percent bootstrap (1000) values.

Genetic relationship among populations was further confirmed by the construction of phylogenetic tree (NJ), using Nei’s genetic distance (Fig. 3). Tibetan sheep population was most distinct, and separated first. Results illustrated clustering together of geographically close sheep breeds, while Tibetan, Changthangi and Bonpala were placed on separate nodes.

The overall accuracy of population self-assignment was very high (Supplementary Table S3), to the tune of 96%. All the animals, except one of Rampur Bushair, Bandur, and Balangir, seven of Hassan and nine of Bellary breeds were correctly assigned to their respective groups. Clustering using Bayesian approaches was performed on the entire data set, with an increasing number of inferred clusters (K) from 2 to 13. The likely value of K, which best captures the variation present in the data was seven (Supplementary Fig. S2). Changthangi, Bonpala, Tibetan, and Shahabadi formed their clusters. Rampur Bushair and Poonchi animals of NT were partitioned into one cluster. Similarly, breeds of the SP region showed intermixing. Even if K was increased up to 13, individuals of Bellary and Hassan remained admixed.

## Discussion

Evolution is the foundation of the enormous variation existing between and within species as well as between and within breeds of a species. Evolution corresponds to the differences in allele frequencies over time, and these differences can be used for the characterization of populations^5^. Accordingly, the genetic variability of different indigenous sheep breeds in India was investigated.

Molecular diversity studies of farm animal genetic resources using mitochondrial DNA have extensively traced domestication events and proposed multiphyletic origins across different species, including sheep. This study explored the membership of 11 breeds of Indian sheep to the 5 well-defined sheep haplogroups identified across continents^18^. Three highly diverged haplogroups (A, B, and C) including 84.68%, 13.96%, and 1.35% samples, respectively, were evident in the investigated sheep populations. Haplogroups D and E, which are considered rare globally, were not observed in this study. Our results are in agreement with the previous studies in Indian sheep, involving breeds other than those considered for the present investigation, wherein haplogroup A was observed in 89.6% and haplogroup B in 10.38% of the total samples^19^. Five breeds of NWASA region namely Marwari, Nali, Kheri, Muzaffarnagri, and Sonadi were components of clade B in the phylogenetic analysis^19^. Subsequently, haplogroup B was identified in four more breeds (Chokla, Jaisalmeri, Patanwadi, and Garole), representing 15% of total samples. Additionally, Haplogroup C was observed at low frequency (< 1%) in Nali and Jaisalmeri sheep, which are again the breeds of NWASA region^20^. Interestingly, haplogroup B and C were observed for the first time in the other three major agro-ecological zones of India, in the present study. Our observations of identification of three haplogroups in Indian sheep are in concordance with many international studies on mtDNA that have registered the presence of haplogroups A and B in Asia and predominance of haplogroup B in Europe^16,18,25,26^. Although haplogroup C is less frequent, however, its presence has been recorded in Turkey, Portugal, the Caucasus, and China^25^. In Tibetan sheep from China, major haplogroups that have been identified are A, B, and C. However, one animal out of a total of 636 sheep investigated also represented haplogroup D^26^. Despite the predominance of haplogroup A in India, the presence of 2 more haplogroups (B and C) in all the major agro-ecological regions of India suggests introgression due to gene flow between breeds of Asia and Europe.

The average haplotype diversity value observed in the present investigation was 0.850, which was intermediate of the previous two attempts that have explored the mitochondrial DNA diversity in Indian sheep (0.603 and 0.987), but the nucleotide value was higher than either of these studies^19,20^. The number of haplotypes ranged from 6–14 across different breeds, and each breed also had its unique haplotypes. All these statistics point towards high maternal genetic diversity in the populations investigated and also substantiates that the correct sampling strategy of including unrelated animals was followed. Signature of population expansion was evident based on Fu’s Fs, Tajima’s D, goodness-of-fit indices, and mismatch distribution assessed under sudden expansion model in sheep breeds from all the four agro-ecological zones. Mismatch distribution analysis revealed a unimodal distribution which suggests that the populations have undergone a rapid expansion in size. Fu’s *Fs* test which is based on haplotype frequencies as well as Tajima’s *D* test that relies on the difference between the number of polymorphic sites, and the mean number of nucleotide pairwise differences, also suggested expanding populations in all the major zones and whole of India. Despite vast sheep genetic diversity and huge population, lack of political will and financial support have resulted in poor coverage of indigenous sheep under genetic improvement programs. Therefore, it has not been possible to curtail mating between breeds in neighboring areas because of shared pastures. This inference was also supported by the presence of a large number of haplotypes that differed from the most common haplotype by few variations only.

Based on the microsatellite diversity and differentiation, Indian sheep breeds had sufficient diversity, as evidenced by the number of alleles and heterozygosity per breed. Except for Tibetan and Bonpala, the mean observed number of alleles (Na) across other breeds was more than 8. Similarly, excluding Tibetan, mean observed heterozygosity (Ho) was more than 0.6 in all the populations (Table 4). Genetic variation of similar magnitude, in terms of allele diversity (Na, 6.04–9.95) and observed heterozygosity (Ho, 0.62–0.78), has been reported in 17 Indian sheep breeds^22^. Estimates were also comparable with that of the Kajali sheep, that belong to the NWASA region^21^ and four sheep populations of the SP region; Madras Red, Mecheri, and Pattanam^27^ and Tiruchi Black^28^. The genetic variability observed in this study was higher than those reported in Jordan sheep^29^, and sheep breeds of the neighboring country, Pakistan^30^. However, it was comparable to the values reported across the world, for sheep^31,32^. Much higher diversity indices were recorded in Turkish sheep breeds that can be attributed to Turkey being considered as a major sheep domestication center^33^.

The lowest diversity parameters (Na, 5.96; Ho, 0.52) were observed in the Tibetan sheep which may be resulting from its extremely small population size. It is an endangered sheep breed with only 215 animal left^8^. Genetic variation of similar magnitude (0.46–0.55) has also been reported in the endangered Namaqua Afrikaner sheep, indigenous to South Africa^34^, whereas, higher heterozygosity has been reported in the two endangered Spanish breeds, Churratensina (0.66) and Churralebrijana (0.67), despite their small population size^35^. It should be mentioned here that the comparison of diversity estimates should be considered as a suggestive indication of diversity since microsatellite loci used across multiple studies are not uniform. A significant positive F_IS_ value was observed for all the breeds, except very small negative values in Poonchi and Bonpala (Table 4), thereby suggesting a lack of heterozygote in 9 breeds. The highest deficiency (23.4%) was encountered in Tibetan sheep that pointed towards the possibility of inbreeding. However, this could also be attributed to the limited samples or to a shared ancestry of the few samples, which otherwise have been treated as unrelated. A reasonably high F_IS_ value could also be an outcome of sex-biased dispersal, a characteristic of a dwindling population or a consequence of the breeding system. Such an elevated level of inbreeding is a risky proposition as it could lead to genetic diseases. Moreover, it can negatively affect animal health. Higher heterozygote deficiency is generally observed in Indian sheep breeds^15,22,36^. Results for Poonchi (− 0.007) and Bonpala (− 0.021) can be interpreted as possible signs of outbreeding, most likely due to a recent admixture of two (or more) populations.

Genetic relationship and differentiation in the 11 Indian sheep breeds were found to be correlated with the geographic distance, shared ancestral variation, and historical gene flow, according to the multiple approaches employed in this study. Populations depicted a moderate, but significant genetic differentiation (F_ST_ = 0.129 ± 0.012). The Tibetan breed appeared as a distinct breed separated from the others. These results reflected that the within-breed genetic variation was high (87.1%) and this variation could be a valuable tool for genetic improvement and conservation of Indian sheep. Genetic differentiation of similar magnitude (11.1%) has been documented in some other indigenous sheep breeds^22^. F_ST_ values of the examined Indian sheep breeds were comparable to those reported for Baltic, Chiapas, and Akkarman sheep^33^. However, it was much higher than the F_ST_ values reported for several others like Spanish, European, Middle Eastern, Colombian, Pakistani, and Romanian sheep breeds^30,32,37^. Much lower differentiation (5.6–5.9%) has been reported in Indian sheep breeds of SP region^15,27^. In the present study also, the breeds of SP region were less differentiated. Bellary and Hassan were least differentiated among them which may be attributed to the considerable exchange of germplasm among the two populations (Nm = 22.55). Differentiation can be an outcome of geography and reproductive isolation, genetic drift, and founder effect, different production goals, and preferences for the specific traits. In the present study, overall moderate genetic differentiation could be due to the fact that breeds were spread across three agro-ecological zones and larger geographical distances, in India. Similarly, visualization of breed relationship through NJ tree showed the clustering of the breeds, mainly in congruence with their geographic location (Fig. 3).

**Figure 4:**
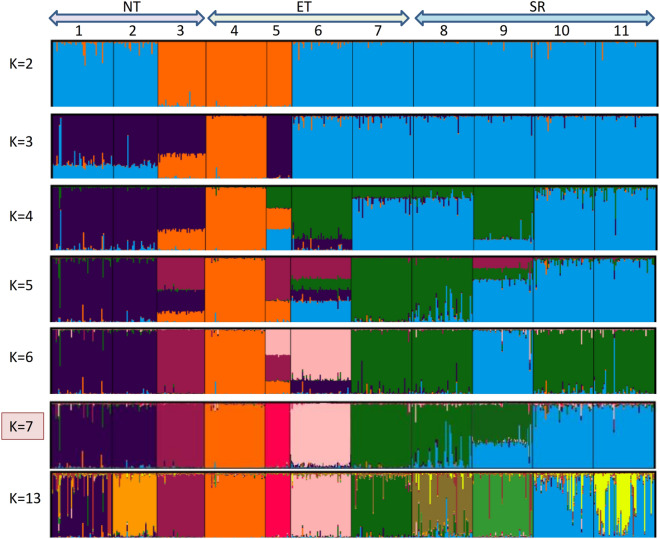
Bayesian clustering of Indian sheep populations under assumption of K = 2–7, and K = 13 clusters using STRUCTURE 2.3.4 program^53^. Each individual is represented by a vertical bar displaying membership coefficients for each genetic cluster. Populations are separated by black lines and denoted by numbers (1 = Rampur Bushair, 2 = Poonchi, 3 = Changthangi, 4 = Bonpala, 5 = Tibetan, 6 = Shahbadi, 7 = Balangir, 8 = Kenguri, 9 = Bandur, 10 = Hassan, 11 = Bellary). Graphics were obtained with DISTRUCT^55^ (https://rosenberglab.stanford.edu/) and CLUMPP^54^ (https://web.stanford.edu/ programs).

The phylogenetic relationship was substantiated by the cluster analyses. The results of STRUCTURE (Fig. 4) support the proposition that Changthangi, Bonpala, Tibetan, and Shahbadi are genetically distinct breeds, while Rampur Bushair and Poonchi are admixed sheep populations. Similarly, all the four sheep breeds of the SP region had indications of gene flow among them. Distinct clusters were most likely the result of different geographic distributions.

Management practices, ecological factors, lack of breeding policies, and geographic continuity in the absence of prominent natural barriers, might have resulted in intermixing among some of the breeds. Sheep management and husbandry practices vary from temperate to tropical ecology of India. Sheep production in the country is largely a nomadic proposition. It is either a pastoral system, where farmers move from place to place along with their flocks or an extensive system in which flocks graze in community land, pastures, and harvested fields, in nearby areas. The intensive scientific production system is restricted only to organized and government farms. The migratory system is the cornerstone of sheep farming in the NT region that falls under the influence of the Himalayas. This migration may be of short duration to neighboring locations or permanent, to long distances over extensive areas. In the plains of the ET region, most of the flocks are stationary and in its Himalayan region, flocks are migratory. Flocks graze on foothills and in the valleys in winter and move to high altitude forests and alpine meadows in summers. In the SP and NWASA regions, migration takes place during the drought conditions in search of foraging and drinking water resources.

Rampur Bushair and Poonchi of the NT region cluster in a single group. This observation is very well supported by the past and prevailing management practices, as well as, the breeding policies. Firstly, shepherds regularly or seasonally migrate with their flocks in search of grazing areas and pasture lands, causing intermixing among the animals. Secondly, most of the animals in this area have been involved in cross-breeding with exotic fine wool breeds (Merino, Rambouillet), for increasing apparel wool production with the concomitant dilution of these breeds. Geographic continuity, ecological similarity, and cultural connections amongst the major sheep rearing communities of the SP region could be contributing towards the gene flow between different sheep populations.

Following the results, conservation programs need to be undertaken for endangered sheep breeds that are threatened with extinction. These include Changthangi, Rampur Bushair, Poonchi, Bonpala, and Tibetan^4^. Tibetan and Bonpala should be prioritized for conservation. These two unique gene pools have low genetic diversity. It is worth mentioning that maternal lineage B dominates in Tibetan sheep, unlike other Indian sheep breeds. Lineage C in Tibetan and Bonpala pointed towards their distinct evolutionary history from other Indian sheep breeds. Similarly, Changthangi represents a unique gene pool with good adaptation to the extremely cold and low oxygen conditions. These animals thrive in the high-altitude cold desert, where, the average altitude of the area is around 14,600 m above sea level. Habitat of Tibetan, Bonpala, and Changthangi is common across the Tibetan plateau^38^. Tibetan and Bonpala sheep migrated to the Eastern agro-ecological region of India with the Tibetan traders, who used them as beasts of burden for transporting various merchandise^39^. They might share ancestral variation and historical gene flow with the sheep breeds of the high-altitude Himalayan region of China. All the three temperate breeds might carry unique adaptation alleles, therefore, these should be considered as a priority group for conservation. Moreover, sustainable breeding programs and policies are required to prevent indiscriminate gene flow among populations and regions, as well as, crossbreeding with the exotic sheep breeds.

Animal breeding farms and research institutes can play a pivotal role in the planning and execution of conservation programs. The establishment of breed associations and nucleus breeding farms is the first step towards conservation and sustainable utilization of indigenous sheep resources. Frequent exchange of the rams among farms and flocks of the same breed needs to be in place to increase the genetic diversity. Incentives to livestock keepers, extension, and health services to migratory flocks, availability of quality breeding rams for each breed, and community involvement can contribute enormously to this endeavor.

## Conclusion

The findings of this study contribute to the knowledge of the genetic diversity and the structure of native Indian sheep breeds. Data generated could be a useful source for breeding management to decrease heterozygote deficiency and breed dilution, and to ensure sustainable management and conservation activities to preserve sheep's genetic heritage. Overall, Tibetan sheep emerged out to be the most interesting gene pool, from both microsatellite and mitochondrial analysis. Genetic diversity indices warrant immediate scientific management and conservation of this invaluable germplasm, that is considered endangered at the moment.

## Materials and methods

### Animals

In total, 477 animals of 11 breeds (Rampur Bushair, Poonchi, Changthangi, Bonpala, Tibetan, Shahbadi, Balangir, Kenguri, Bandur (Mandya), Hassan and Bellary) were sampled after field survey in their respective distribution areas, spread across Northern, Eastern and Southern India (Fig. 1). Animals included in this study represented the original autochthonous phenotype. Guidelines of Measurement of Domestic Animal Diversity program^10^ were followed in selecting animals. For each breed, blood samples were collected from unrelated sheep belonging to multiple flocks, and across different villages. Farmers were interviewed in detail to ascertain the unrelatedness of collected samples. Blood sampling was carried out between 2015 and 2019. All the 477 samples were utilized for the microsatellite analysis, whereas the first 20–22 samples of each breed were used for the mitochondrial study. Genomic DNA was extracted from blood using standard phenol–chloroform protocol^40^. The quantity of DNA was analyzed using a NanoDrop 1000 spectrophotometer (Thermo Scientific).

### Ethics statement

Sheep blood (~ 5 ml) was aseptically collected by the trained veterinarians from the jugular vein with the consent of the farmers. The study has the approval of the Institute Animal Ethics Committee of Indian Council of Agricultural Research, National Bureau of Animal Genetic Resources, Karnal (PME Ref. 11/ 2017-18), and their relevant guidelines were followed during blood sample collection.

### Amplification and sequencing of mtDNA

DNA samples from all 11 breeds were amplified using previously reported primers and cycling conditions^41^. Primers, F-AACTGCTTGACCGTACATAGTA, and R-AGAAGGGTATAAAGCACCGCC amplified a 1,246 bp fragment that spans part of the control region, the tRNA-Phe and 12S rRNA coding RNA genes. The amplified fragments were enzymatically purified using Exonuclease I and Antarctic Phosphatase (New England Biolab, USA), followed by sequencing in ABI 3100 Automated DNA Sequencer (Applied Biosystems, USA) using Big Dye Terminator Cycle Sequencing Kit (Applied Biosystems, USA).

### Analytical methodologies for mtDNA

The amplified sequences were aligned using the Clustal W algorithm embedded in MEGA version 6.0^42^. The genetic diversity indices such as the number of haplotypes, haplotype diversity, nucleotide diversity, and the number of polymorphic sites for each breed and across all breeds were estimated using DnaSP v5^43^. The analysis of molecular variance within and among agro-ecological zones was done using the AMOVA program implemented in the Arlequin version 3.5^44^. To get a comprehensive insight into the phylogenetic relationship of Indian sheep in the context of global ovine diversity, sequences from previous studies in Indian sheep^19^ and 10 reference sequences for the 5 known sheep haplogroups identified across the globe were retrieved from NCBI, and a meta-analysis was carried out with the sequences of the present study. The purpose of this exhaustive analysis was to include breeds that represent all the four major agro-ecological regions of India. Departures from neutrality using Fu’s Fs^45^ and Tajima’s D statistics^46^, demographic changes based on Harpending's raggedness index, and the sum of squared deviations (SSD) between the observed and expected mismatch, as well as, population pairwise F_ST_ were examined using Arlequin version 3.5^44^. Reference sequences of the 5 known sheep mitochondrial haplogroups were retrieved from NCBI, as per the assignment given by Meadows et al.^18^: Haplogroup A: DQ852105 and DQ852113; Haplogroup B: DQ852157 and DQ852189; Haplogroup C: DQ852252 and DQ852260; Haplogroup D: DQ852274 and DQ852275; and Haplogroup E: DQ852276 and DQ852278. These sequences were included along with the sequences obtained in this study for constructing a Median-joining (MJ) network using NETWORK 10.0.0.0^47^, and Neighbour-joining (NJ) tree using MEGA6, for exploring the association between the haplotypes.

### Nuclear microsatellite genotyping

Twenty-five International Society of Animal Genetics and FAO-recommended sheep nuclear microsatellites were analyzed in all the individual animals^10^. These simple sequence repeat markers (nSSR) were highly polymorphic, spread all over the genome, and with the ability to co-amplify in PCR reactions (Supplementary Table S4). The workflow consisted of (1) amplification using a fluorescent dye (FAM, HEX, NED and PET) labeled primers, as described by Sharma et al.^8^ (2) multiplex genotyping using an ABI 3100 Automated DNA Sequencer (Applied Biosystems, USA) along with the internal line control (3) electropherogram analysis using GeneMapper software (Applied Biosystems, USA) for allele size estimation, and (4) statistical analysis using different software packages.

### Data analysis for nSSR

Allelic polymorphisms at each nSSR locus were evaluated. The program MICRO-CHECKER^48^ was used to examine large allele dropout, stuttering, and null alleles as potential sources of error. The genotype data were analyzed using GenAlEx 6.5 software^49^ to calculate allele frequencies at each locus for each population, the average number of allele per population; observed (Na) and effective numbers of alleles (Ne) and heterozygosity values (observed, Ho and expected, He, Shannon information index (I) and heterozygote deficit (F_IS_) per locus across breeds and markers. Average values were expressed as Mean ± SE from values at each locus. Chi-square tests of deviations from Hardy–Weinberg equilibrium (HWE) were derived.

To study the relationships and the genetic differentiation among tested populations, different F statistics estimates^50^, analysis of molecular variance (AMOVA), and genetic distances were applied. Nei’s genetic distance^51^ and Cavalli-Sforza Chord distance were estimated by GenAlEx 6.5^49^. Statistical significance of F estimates was tested using FSTAT, version 2.9.3 software^52^. The dendrograms of phylogenetic trees were built from different distance matrices and were visualized by MEGA6^42^ using the NJ approach. Genotypic disequilibrium was tested by using FSTAT 2.9.3^52^ for all pairs of loci with 1,000 permutations. Population assignment was performed using multilocus genotypes of individuals. STRUCTURE ver 2.3.4 software^53^ was used to further assess the degree of population differentiation within and between the 11 populations. Individuals were assigned into clusters using the Bayesian method under an admixture model. The algorithm estimates allele frequencies for each gene pool (cluster) and population memberships for every individual. The Simulation was run ten times for each value of K (2–13), where K is the number of tested clusters. All runs were performed with a 300,000 burnin period and 500,000 MCMC repeats after burnin. Software CLUMPP^54^ was used to align multiple replicates for each K to facilitate the interpretation of clustering results. The DISTRUCT application^55^ was used to graphically display the results. To determine the optimal number of groups (K), we utilized both the log-likelihood method of Prichard et al.^56^ as well as Δ*K* value of Evanno et al.^57^ and was calculated and plotted using Structure Harvester application^58^.

## Supplementary information

Supplementary Information.

## Data Availability

Sequences of unique mitochondrial DNA haplotypes of each sheep breed were submitted in GenBank (Accession numbers: MK061173 - MK061283).
