# Supplementary material for: Microsatellite and mitochondrial DNA analyses unveil the genetic structure of native sheep breeds from three major agro-ecological regions of India
Source: Sci Rep. 2020 Nov 24;10:20422. doi: 10.1038/s41598-020-77480-6 (PMC7687881; doi:10.1038/s41598-020-77480-6)
Supplement: Supplementary file 1 — Supplementary Information. [file 41598_2020_77480_MOESM1_ESM.docx]

**Microsatellite and mitochondrial DNA analyses unveil the genetic structure of native sheep breeds from three major agro-ecological regions of India**

Rekha Sharma*, Sonika Ahlawat, Himani Sharma, Priyanka Sharma, Poonam Panchal, Reena Arora and MS Tantia

ICAR-National Bureau of Animal Genetic Resources, Karnal

Email: [Rekha.Sharma@icar.gov.in](mailto:Rekha.Sharma@icar.gov.in), rekvik@gmail.com

**SUPPLEMENTARY INFORMATION**

**
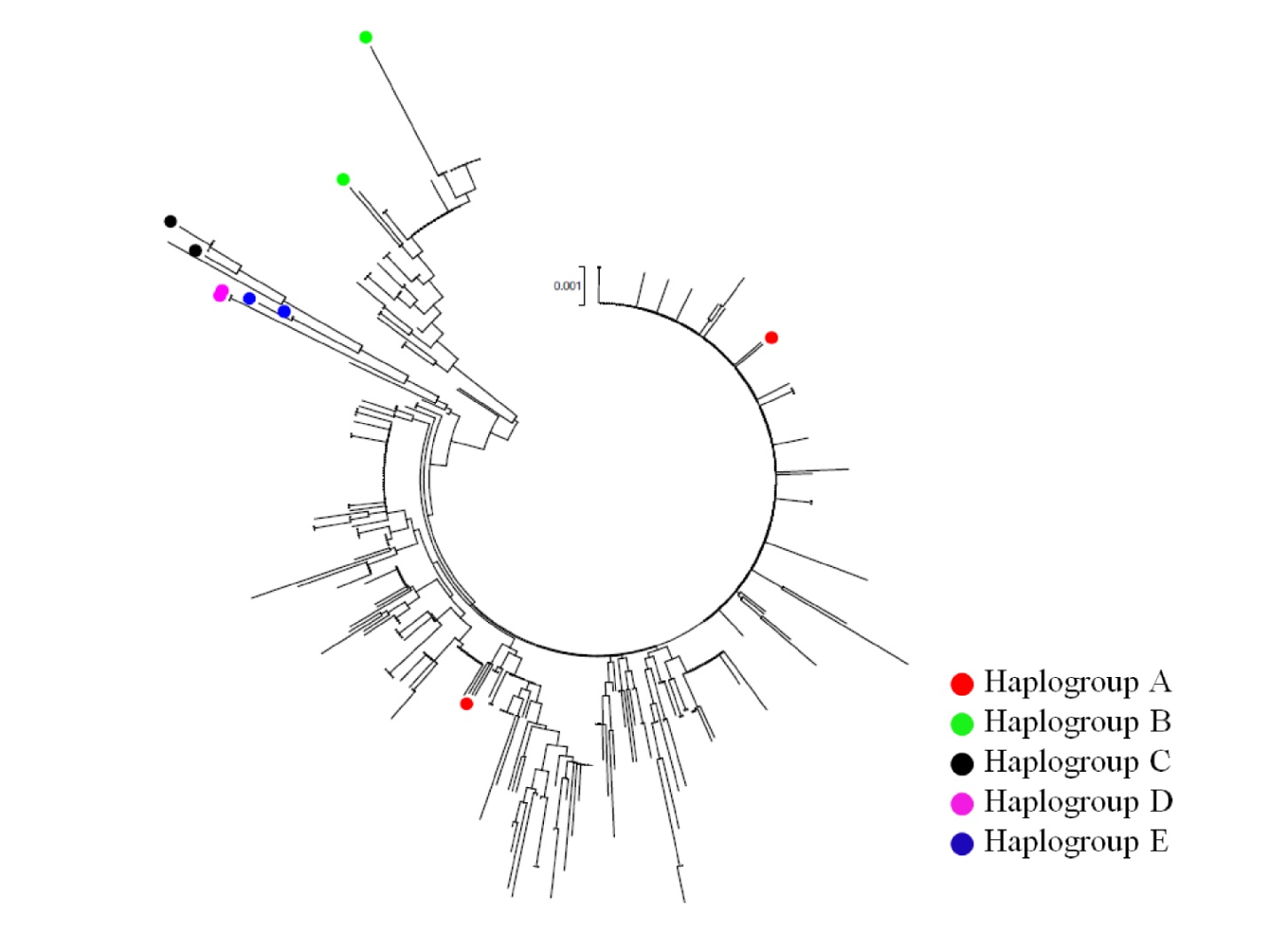
**

**Figure S1.** A neighbor joining phylogenetic tree of 11 Indian sheep breeds and representatives of 5 known haplogroups using MEGA 6^42^ (https://www.megasoftware.net/).

**Table S1.** F-Statistics and estimates of Nm for all populations and loci

| Locus | Fis | Fit | Fst | Nm |
| --- | --- | --- | --- | --- |
| BM0757 | 0.087 | 0.197 | 0.121 | 1.815 |
| BM0827 | 0.052 | 0.146 | 0.099 | 2.278 |
| BM1314 | 0.175 | 0.282 | 0.129 | 1.686 |
| BM6506 | 0.040 | 0.274 | 0.244 | 0.776 |
| BM6526 | -0.007 | 0.115 | 0.121 | 1.817 |
| BM8125 | 0.053 | 0.127 | 0.078 | 2.949 |
| CSRD247 | 0.066 | 0.187 | 0.129 | 1.681 |
| CSSM31 | 0.103 | 0.220 | 0.130 | 1.667 |
| CSSM47 | 0.250 | 0.513 | 0.351 | 0.463 |
| HSC | 0.066 | 0.131 | 0.069 | 3.363 |
| INRA63 | -0.010 | 0.059 | 0.068 | 3.415 |
| MAF214 | 0.097 | 0.217 | 0.132 | 1.640 |
| OarAE129 | 0.229 | 0.374 | 0.188 | 1.080 |
| OarCP20 | -0.098 | 0.047 | 0.132 | 1.649 |
| OarCP34 | 0.109 | 0.198 | 0.100 | 2.261 |
| OarCP49 | 0.038 | 0.133 | 0.099 | 2.275 |
| OarFCB128 | -0.007 | 0.128 | 0.134 | 1.623 |
| OarFCB48 | 0.157 | 0.234 | 0.091 | 2.486 |
| OarHH35 | 0.075 | 0.195 | 0.130 | 1.667 |
| OarHH41 | 0.099 | 0.184 | 0.095 | 2.387 |
| OarHH47 | 0.019 | 0.081 | 0.063 | 3.728 |
| OarHH64 | 0.372 | 0.488 | 0.186 | 1.096 |
| OarJMP029 | -0.010 | 0.093 | 0.102 | 2.200 |
| OarJMP08 | 0.050 | 0.145 | 0.100 | 2.249 |
| OarVH72 | 0.109 | 0.229 | 0.134 | 1.614 |
| Mean | 0.085 | 0.200 | 0.129 | 1.995 |
| SE | 0.019 | 0.023 | 0.012 | 0.158 |

**Table S2.** Results of analysis of molecular variance (AMOVA) test in different populations of Indian sheep. ***P < 0.001

| Source of variation | Sum of squares | Estimated variance | Percentage variation | F statistics | Nm |
| --- | --- | --- | --- | --- | --- |
| Among populations | 1084.403 | 1.137 | 11 | Fst= 0.111*** | 2.010 |
| Among individuals within populations | 4781.785 | 1.125 | 11 | Fis= 0.123*** |  |
| Within individuals | 3821.000 | 8.010 | 78 | Fit= 0.220*** |  |
| Total | 9687.188 | 10.272 | 100 |  |  |

**Table** **S3.** Summary of population assignment outcomes to 'self' or 'other' population (With leave one out option)

| **Region** | **Population** | **Self population** | **Other population** |
| --- | --- | --- | --- |
| NT | Rampur Bushair | 47 | 1 |
|  | Poonchi | 35 |  |
|  | Changthangi | 38 |  |
| ET | Bonpala | 48 |  |
|  | Tibetan | 20 |  |
|  | Shahbadi | 48 |  |
|  | Balangir | 47 | 1 |
| SP | Kenguri | 48 |  |
|  | Bandur | 47 | 1 |
|  | Hassan | 41 | 7 |
|  | Bellary | 39 | 9 |
|  | Total | 458 | 19 |
|  | Percent | 96 | 4 |


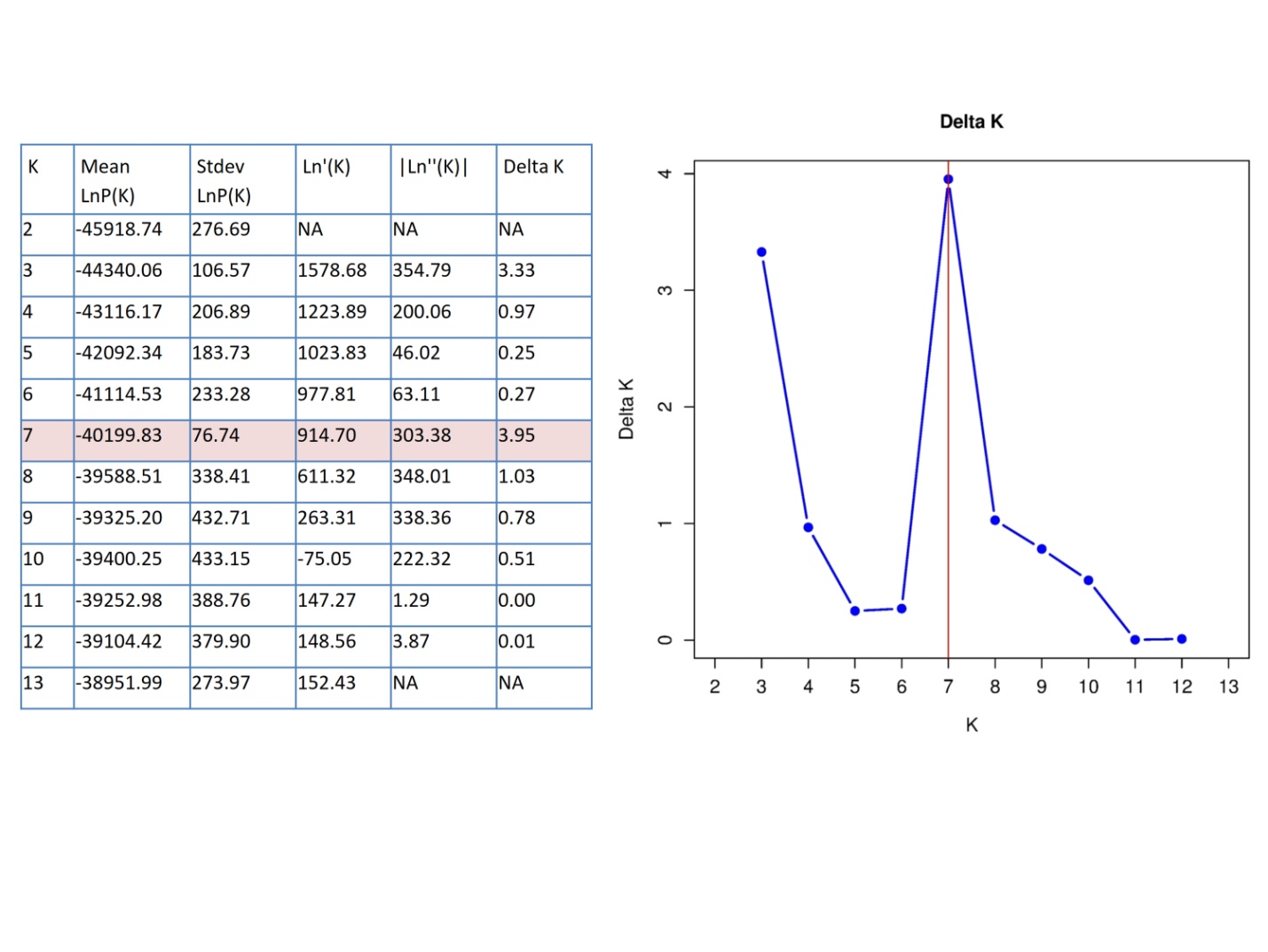


**Figure S2.** Selection of appropriate K value (Evanno method) by analyzing the output of STRUCTURE 2.3.4 software using the Structure Harvester^58^ program (http://taylor0.biology.ucla.edu/). Congruent value (marked with red line) is observed at K = 7.

**Table S4.** Details of primers (sequence, fluorescent dye, type of repeat, size range, location, and accession numbers) used for nSSR markers

| **Panel** | **Locus** | **Primer sequence**  (5′ → 3′) | **Dye** | **Type of repeat** | **Observed size range**  **(bp)** | **Chr no.** | **GenBank Acc. no.** |
| --- | --- | --- | --- | --- | --- | --- | --- |
| Panel 1 | BM757 | Tggaaacaatgtaaacctggg  ttgagccaccaaggaacc | FAM | (GT)_17_ | 178-226 | 9 | G18473 |
|  | BM8125 | Ctctatctgtggaaaaggtggg  gggggttagacttcaacatacg | FAM | - | 93-131 | 17 | G18475 |
|  | OarHH47 | Tttattgacaaactctcttcctaactccacc  gtagttatttaaaaaaatatcatacctcttaagg | VIC | (AC)_32_ | 110-148 | 18 | L12557 |
|  | BM827 | Gggctggtcgtatgctgag  gttggacttgctgaagtgacc | NED | - | 196-230 | 3 | U06763 |
|  | OarCP49 | Cagacacggcttagcaactaaacgc  gtggggatgaatattccttcataagg | NED | (AC)_17_ | 62-160 | 17 | U15702 |
| Panel 2 | CSSM47 | Tctctgtctctatcactatatggc  ctgggcacctgaaactatcatcat | VIC | (TG)_12_TATGTA(TG)_4_ | 116-168 | 2 | U03821 |
|  | OarHH41 | Tccacaggcttaaatctatatagcaacc  ccagctaaagataaaagatgatgtgggag | NED | (AC)_23_ | 116-160 | 10 | L12555 |
|  | OarVH72 | Ctctagaggatctggaatgcaaagctc  ggcctctcaaggggcaagagcagg | FAM | (AC)_14_ | 119-137 | 25 | L12548 |
|  | OarCP20 | Gatcccctggaggaggaaacgg  ggcatttcatggctt tagcagg | PET | (AC)_14_ | 57-103 | 21 | U15695 |
|  | MAF214 | Aatgcaggagatctgaggcagggacg  gggtgatcttagggaggttttggagg | PET | - | 156-275 | 16 | M88160 |
| Panel 3 | BM6526 | Catgccaaacaatatccagc  tgaaggtagagagcaagcagc | VIC | - | 130-186 | 26 | G18454 |
|  | OarCP34 | Gctgaacaatgtgatatgttcagg  gggacaatactgtcttagatgctgc | FAM | (AC)_17_TTGCGTGT(CA)_4_ | 94-142 | 3 | U15699 |
|  | OarAE129 | Aatccagtgtgtgaaagactaatccag  gtagatcaagatatagaatatttttcaacacc | NED | (AC)_14_ | 115 -179 | 5 | L11051 |
|  | OarFCB128 | Cagctgagcaactaagacatacatgcg  attaaagcatcttctctttatttcctcgc | PET | (GT)_6_GC(GT)_15_ | 79-115 | 2 | L01532 |
|  | INRA63 | Gaccacaaagggatttgcacaagc  aaaccacagaaatgcttggaag | FAM | (AC)_13_ | 159-211 | 14 | X71507 |
| Panel 4 | OarHH35 | Aattgcattcagtatctttaacatctggc  atgaaaatataaagagaatgaaccacacgg | NED | (TG)_17_ | 111-169 | 4 | L12554 |
|  | OarHH64 | Cgttccctcactatggaaagttatatatgc  cactctattgtaagaatttgaatgagagc | PET | (TG)_17_ | 110-136 | 4 | L12558 |
|  | OarJMP8 | Cgggatgatcttctgtccaaatatgc  catttgctttggcttcagaaccagag | VIC | (GT)_n_ | 107-173 | 6 | U35059 |
|  | OarJMP29 | Gtatacacgtggacaccgctttgtac  gaagtggcaagattcagaggggaag | FAM | (CA)_21_ | 78-178 | 24 | U30893 |
|  | HSC | Ctgccaatgcagagacacaaga  gtctgtctcctgtcttgtcatc | FAM | - | 243-295 | 20 | M90759 |
| Panel 5 | BM1314 | Ttcctcctcttctctccaaac  atctcaaacgccagtgtg g | NED | - | 125-185 | 22 | G18433 |
|  | CSSM31 | Ccaagtttagtacttgtaagtaga  gactctctagcactttatctgtgt | VIC | AAAA(CA)_7_TA(CA)_25_ | 120-192 | 23 | U03838 |
|  | BM6506 | Gcacgtggtaaagagatggc  agcaacttgagcatggcac | FAM | - | 175-203 | 1 | G18455 |
|  | OarFCB48 | Gagttagtacaaggatgacaagag gcac  gactctagaggatcgcaaagaaccag | PET | (TG)_11_CA(TG)_3_ | 128-246 | 17 | M82875 |
|  | CSRD247 | Ggacttgccagaactctgcaat  cactgtggtttgtattagtcagg | NED | (AC)_n_ | 191-267 | 14 | EU009450 |
